# Supplementary material for: Information uncertainty influences learning strategy from sequentially delayed rewards
Source: PLoS Comput Biol. 2026 Feb 2;22(2):e1013879. doi: 10.1371/journal.pcbi.1013879 (PMC12885371; doi:10.1371/journal.pcbi.1013879)
Supplement: S1 Table — Six additional models were tested in relation to our hybrid model using Akaike information criterion (AIC) as our model comparison metric: (i) an eligibility-tabular hybrid with a shared learning rate (Hybrid-1L) between both models; (ii) an eligibility-tabular hybrid with a shared learning rate and shared decay rate (Hybrid-1D) between both models; (iii) an eligibility model with fixed learning and decay rates (Eligibility-Fix) using the mean best fitting parameters from the eligibility model; (iv) a tabular model with fixed learning and decay rates (Tabular-Fix) using the mean best fitting parameters from the tabular model; and (v) a fixed hybrid model (Hybrid-Fix), using the mean best fitting parameters from the hybrid model. We compared all models using a paired Wilcoxon signed-rank tests to the best-fitting (indicated by -) model in each condition. Models that did not significantly differ from the best-fitting model (bolded) represent statistically equivalent alternatives. However, most shared parameter models (1L and 1D) showed similar results to the full hybrid model. (DOCX) [file pcbi.1013879.s010.docx]

**S1 Table. Additional model comparisons.**

|  | Group: Disjoint → Conjoint (N=74) | | | | Group: Conjoint → Disjoint  (N=68) | | | |
| --- | --- | --- | --- | --- | --- | --- | --- | --- |
|  | Disjoint-1 | | Conjoint-2 | | Conjoint-1 | | Disjoint-2 | |
|  | AIC | *p* | AIC | *p* | AIC | *p* | AIC | *p* |
| Eligibility | 360 | .001 | **360.9** | **.46** | **391.5** | **.08** | **391** | **.14** |
| Eligibility-Fix | 376.2 | .001 | 383.3 | <.001 | 410.8 | <.001 | 407.8 | .001 |
| Tabular | 357.5 | .001 | 383.2 | .001 | 410.9 | <.001 | 400.2 | <.001 |
| Tabular-Fix | 364 | .001 | 392.6 | <.001 | 417.2 | <.001 | 406.7 | <.001 |
| Hybrid | **342.5** | - | **358.3** | **.14** | **391.3** | - | 383.7 | .02 |
| Hybrid-1L | **343.8** | **<.21** | 359.3 | <.001 | **391.4** | **.4** | 384.2 | <.001 |
| Hybrid-1D | **343.7** | **<.33** | **358** | - | 390.1 | .02 | **383.3** | - |
| Hybrid-Fix | 354.9 | .001 | 379.6 | <.001 | 408.7 | <.001 | 398.4 | <.001 |

Six additional models were tested in relation to our hybrid model using Akaike information criterion (AIC) as our model comparison metric: (i) an eligibility-tabular hybrid with a shared learning rate (Hybrid-1L) between both models; (ii) an eligibility-tabular hybrid with a shared learning rate and shared decay rate (Hybrid-1D) between both models; (iii) an eligibility model with fixed learning and decay rates (Eligibility-Fix) using the mean best fitting parameters from the eligibility model; (iv) a tabular model with fixed learning and decay rates (Tabular-Fix) using the mean best fitting parameters from the tabular model; and (v) a fixed hybrid model (Hybrid-Fix), using the mean best fitting parameters from the hybrid model. We compared all models using a paired Wilcoxon signed-rank tests to the best-fitting (indicated by -) model in each condition. Models that did not significantly differ from the best-fitting model (bolded) represent statistically equivalent alternatives. However, most shared parameter models (1L and 1D) showed similar results to the full hybrid model.
