# Supplementary material for: Information uncertainty influences learning strategy from sequentially delayed rewards
Source: PLoS Comput Biol. 2026 Feb 2;22(2):e1013879. doi: 10.1371/journal.pcbi.1013879 (PMC12885371; doi:10.1371/journal.pcbi.1013879)
Supplement: S2 Table — To test whether our main conclusion is held under different normalization schemes, we compared our theoretically motivated 5-trial scaling window with other scaling schemes (no scaling, 1-, 3- and 7-trial scaling window, and scaling across the entire task). We display AIC values for conjoint and disjoint conditions under two preprocessing approaches: raw value functions (no scaling) versus normalized value functions (5-trial Z-score scaling). The comparison demonstrates that value scaling does not alter model fit patterns, as confirmed by Wilcoxon signed-rank tests comparing conjoint and disjoint conditions for each model and each scaling approach. (DOCX) [file pcbi.1013879.s011.docx]

**S2 Table. Akaike information criterion (AIC) table comparing value normalization schemes.**

| AIC | No scaling | | | | 5-trial Z-score scaling | | | |
| --- | --- | --- | --- | --- | --- | --- | --- | --- |
|  | Conjoint | Disjoint | V | p | Conjoint | Disjoint | V | p |
| Eligibility | 375.22 | 371.39 | 5725 | .19 | 375.55 | 374.86 | 5516 | .37 |
| Tabular | 373.9 | 360.81 | 6190 | .02 | 396.44 | 377.98 | 6647 | .001 |
|  | 1-trial Z-score scaling | | | | 3-trial Z-score scaling | | | |
|  | Conjoint | Disjoint | V | p | Conjoint | Disjoint | V | p |
| Eligibility | 374.94 | 374.21 | 5526 | .36 | 375.34 | 374.61 | 5525 | .36 |
| Tabular | 395.29 | 376.71 | 6637 | .001 | 395.96 | 377.34 | 6655 | .001 |
|  | 7-trial Z-score scaling | | | | 336-trial Z-score scaling | | | |
|  | Conjoint | Disjoint | V | p | Conjoint | Disjoint | V | P |
| Eligibility | 375.73 | 375.06 | 5534 | .35 | 373.52 | 373.81 | 5409 | .5 |
| Tabular | 396.79 | 378.53 | 6633 | .002 | 396.93 | 376.62 | 6701 | <.001 |

To test whether our main conclusion is held under different normalization schemes, we compared our theoretically motivated 5-trial scaling window with other scaling schemes (no scaling, 1-, 3- and 7-trial scaling window, and scaling across the entire task). Displaying Akaike Information Criterion (AIC) values for Conjoint and Disjoint conditions under two preprocessing approaches: raw value functions (no scaling) versus normalized value functions (5-trial Z-score scaling). The comparison demonstrates that value scaling does not alter model fit patterns, as confirmed by Wilcoxon signed-rank tests comparing Conjoint and Disjoint for each model and each scaling approach.
