# Supplementary material for: Information uncertainty influences learning strategy from sequentially delayed rewards
Source: PLoS Comput Biol. 2026 Feb 2;22(2):e1013879. doi: 10.1371/journal.pcbi.1013879 (PMC12885371; doi:10.1371/journal.pcbi.1013879)
Supplement: S3 Table — Pearson’s correlations with diagonals as correlations between disjoint-conjoint, below are conjoint-conjoint correlations and above are disjoint-disjoint correlations. * p < .05, ** p < .01. (DOCX) [file pcbi.1013879.s012.docx]

**S3 Table. Correlation between independent model parameters.**

| Parameter | BetaTab | BetaElg | LambdaTab | LambdaElg | AlphaTab | AlphaElg |
| --- | --- | --- | --- | --- | --- | --- |
| BetaTab | **.45**** | **.82**** | **.21*** | **.27**** | .04 | **-.24**** |
| BetaElg | **.81**** | **.37**** | .12 | .13 | -.1 | **-.4**** |
| LambdaTab | .11 | .03 | .06 | .09 | -.06 | .02 |
| LambdaElg | **.26**** | .07 | .06 | **.18*** | .06 | .03 |
| AlphaTab | **-.33**** | **-.4**** | .02 | -.01 | -.04 | **.25**** |
| AlphaElg | **-.43**** | **-.44**** | -.08 | .02 | **.62**** | **.32**** |

Pearson’s correlations with diagonals as correlations between disjoint-conjoint, below are conjoint-conjoint correlations and above are disjoint-disjoint correlations. * p < .05, ** p < .01
