# Supplementary material for: Information uncertainty influences learning strategy from sequentially delayed rewards
Source: PLoS Comput Biol. 2026 Feb 2;22(2):e1013879. doi: 10.1371/journal.pcbi.1013879 (PMC12885371; doi:10.1371/journal.pcbi.1013879)
Supplement: S1 Text — (DOCX) [file pcbi.1013879.s013.docx]

**Participant Demographics**

Most participants (n = 90) were from Europe (33 Portugal, 30 Poland, 6 Italy, 5 Hungary, 5 United Kingdom, 4 Greece, 4 Spain, and 3 other). The other subjects were predominantly spread across North America (n = 22) and South Africa (n = 22); a total of eight other subjects were in the Middle East and Asia. Although all participants were fluent in English, their first languages were predominantly Portuguese (n = 34), Polish (n = 31), Spanish (n = 25), English (n = 10), Other (n = 22), and missing (n = 20).

**Instruction Questions**

Participants answered four instruction questions to help solidify their understanding (M_correct_ = 56% SD_correct_ = 19%). Upon answering one of the four instruction questions incorrectly, further instruction was provided. No participants were excluded based on instruction questions. At the end of the instructions, the final question asked if they felt they understood the task. Among the participants, 87% reported feeling comfortable and 13% reported feeling slight to moderate confusion. The specific object reward and whether the object had a delayed reward had to be learned throughout the task. Participants were told in the instructions that delayed objects always had a fixed delay of two trials ahead. After each participant completed the CA task, they took an exit survey. Time taken, in minutes, for the CA task (Disjoint: M_end_ = 25.27, SD_end_ = 8.77, Conjoint: M_end_ = 25.32, SD_end_ = 10.07) and surveys (M_end_ = 7.25, SD_end_ = 3.85) were reasonable. Per-trial time was calculated after removing non-answered trials, for an average trial time of 1.59 seconds (SD_trialtime_ = 1.37).

**Parameter Recovery**

To achieve proficient parameter recovery, we simulated 300 datasets using modest uncertainty to generate each of the 300 parameter sets. For each model's learning rate and decay rate, a beta distribution was utilized (α = 1.25, β = 1.25), while the decision weights used a gamma distribution (α = 1.25, β = 1). The three generated parameters were held constant between the two independent models and feedback conditions. Additionally, for the hybrid model, these six generated parameters were held constant across both feedback conditions. Each of the generative models returned the simulated agent’s choice, value functions, and probabilities. Afterwards, the model was optimized using an evolutionary strategy (R package ‘DEoptim’) on the simulated agent’s choices to identify the correlation between generative and recovered parameters (Mullen et al., 2011). To avoid local minima, we set a population size (750 for each model and 1500 for hybrid), a modest number of iterations (60 for each model and 120 for hybrid), and algorithm strategy #3 which provided a jitter to the inherited parameters from the previous fit iteration.

Additionally, priors were used corresponding to the generated parameter distributions with modest uncertainty, and the z-scored locally weighted smoothing function was used. Pearson’s correlations between simulated and recovered parameters were then computed and transposed on a scatterplot with a fitted line (S1 Fig). For eligibility-conjoint, alpha (learning rate), r(299) = .86, beta (decision weight), r(299) = .99, and lambda (decay rate), r(299) = .81. For eligibility-disjoint, alpha, r(299) = .84, beta, r(299) = .98, and lambda, r(299) = .83. For tabular-conjoint, alpha, r(299) = .33, beta, r(299) = .92, and lambda, r(299) = .61. For tabular-disjoint, alpha, r(299) = .64, beta, r(299) = .85, and lambda, r(299) = .64. For hybrid-conjoint, alpha-eligibility, r(299) = .48, beta-eligibility, r(299) = .9, and lambda-eligibility, r(299) = .5, alpha-tabular, r(299) = .33, beta-tabular, r(299) = .84, and lambda-tabular, r(299) = .63. For hybrid-disjoint, alpha-eligibility, r(299) = .59, beta-eligibility, r(299) = .89, and lambda-eligibility, r(299) = .6, alpha-tabular, r(299) = .32, beta-tabular, r(299) = .83, and lambda-tabular, r(299) = .54. Noteworthy, beta-tabular appears to be underfitting the trend, along with beta-eligibility under-weighing and over-weighing based on the reward condition (see S7 Fig).

**Reinforcement Learning Model Parameters Across Conditions**

Examining the correlation between parameters, we found that some of the individual model parameters were correlated with one another (S3 Table). Noteworthy, correlations amongst the parameters in different conditions (between conjoint and disjoint) were surprisingly low, but some were significant. The strongest correlations appeared in the relationship of the strategy weights between eligibility and tabular; however, this did not hold across conditions. The strategy weight also represents the degree of decision stochasticity or reward sensitivity when selecting between two choices and thus correlating between models. Other parameters seemed to trade off depending on the relationship between learning rate, strategy weight, and decay weight.
