# Supplementary material for: Information uncertainty influences learning strategy from sequentially delayed rewards
Source: PLoS Comput Biol. 2026 Feb 2;22(2):e1013879. doi: 10.1371/journal.pcbi.1013879 (PMC12885371; doi:10.1371/journal.pcbi.1013879)
Supplement: Attachment — Submitted filename: Response to Reviewers.docx [file pcbi.1013879.s015.docx]

Dear Reviewers,

Thank you for reviewing our manuscript with our changed title, “Information uncertainty influences learning strategy from sequentially delayed rewards” (PCOMPBIOL-D-25-00171) and for your astute concerns and suggestions. We have taken all of them into account when revising the manuscript and believe the revised manuscript is now considerably improved as a result.

Specifically, we have added substantial analyses to strengthen and validate our modelling approach (as suggested by all Reviewers), including (1) additional analysis to justify some of our claims with revisions to our manuscript’s language (2) extensive posterior predictive check analyses utilizing best-fitting model parameters (Fig 3 & 4) along with additional graphics and interpretation, (3) model recovery analysis using AIC and BIC to determine the better estimator of model fit, and (4) testing additional reinforcement learning models. We have addressed some of the concerns of Reviewer #2 on temporal and information uncertainty along with task difficulty with revisions to our title, manuscript’s language, and additional analysis. Furthermore, we have added additional citations per the reviewer’s suggestions. As pointed out by Reviewer #1, we have also provided additional context on fitting procedures along with clarification on our modeling specifications. We have also provided a section on the justification for the standardization of our value estimates.

For ease of reference, we have added your comments below in bold followed by our responses with page and line number for any changes within the manuscript.

With these revisions, we hope the manuscript will be suitable for publication at *PLoS Computational Biology*.

**Reviewer #1: The paper by Maulhardt, Solway, and Charpentier examined how humans solve the temporal credit assignment when they receive both immediate and delayed rewards. Using computational modeling, the authors found that when participants have no explicit knowledge about the source of each part of the rewards, they used a retrospective algorithm based on eligibility traces to learn the values of stimuli, which leads to rewards being associated with recent actions that did not causally lead to the current reward. However, when they know the values of immediate and delayed rewards respectively, they instead use a prospective tabular algorithm which more accurately associates reward with the correct action. The task design is novel but straightforward and provides important new insight about human reinforcement learning. To enhance the clarity and impact of the results, the modeling approach could benefit from some more elaboration. Some questions and proposed additional analyses are presented below that could strengthen the results and lend more support the current conclusions.**

**1. In the result of Fig. 3, it is unclear whether the models were simulated using each subject’s best-fitting parameters or parameters drawn from some prior distribution. In order to validate these models, it would be best to fit them to behavior first and then simulate using the best-fitting parameters. This applies similarly to Fig. 4.**

We agree with the reviewer and should have been more detailed and explicit with our model-fitting details. We give a more detailed description below and have made revisions to the manuscript to reflect the details of the model-fitting procedures.

For Fig 3., we fit three logistic regressions from equation 1: one on the participants’ data, and two on the data generated from eligibility and tabular models. The data generated from the models indeed used the participants’ best-fitting parameters, as the reviewer suggests. We have now made clear both in the results and in the methods section.

Results (P.11, L.237): “Using equation 1, we conducted three logistic regression analyses: one with the participants' data, and two with a simulated dataset using 25 iterations of each participant’s best-fitting parameters (see Methods for details) for both the eligibility and tabular choice models (Fig 3B).”

Methods, (P.30, L.652): “In our stay-switch signature (equation 1), both eligibility and tabular used the participants’ best fitting parameters to generate model-predicted choice…”

For Fig 4.B, we used the best fitting parameters from the hybrid model to generate choice data, but didn’t elaborate further as this was just for reader visualization. For Fig 4.C (now Fig 4.D), however, we used a broader approach that involved aggregating data across all random walks and pairs, aiming to identify overarching trends and interactions not apparent in individual examples. “To test this, we employed the same task sequences observed by participants and utilized a beta distribution (α = 1, β = 2) for our learning rate, a gamma distribution (α = 3, β = 1) for the inverse temperature parameter, and a beta distribution (α = 2, β = 3) for the decay parameter. This maintained consistent parameters across the two models and kept a high inverse temperature parameter to reduce random noise. ” (P.30, L.310). Without maintaining consistent input for our two models, then systematic differences in choice could be due to differences in parameter input rather than on mechanism, therefore, not using the participants’ best-fitting parameters in this particular analysis provides a stronger argument for model predictability. The two models then made selections on the same sequences seen by participants during the task.

However, given the reviewer’s comment, we decided to try the approach from Fig 3, that is, using model-generated data for 25 repetitions with best fitting parameters. Although the parameter input values are different, the degree of chance might be smoothed away with more repetitions. So, we fit the data from the 25-iteration model-generated dataset with the mixed-effect logistic regression approach from Fig 4D (without maintaining the consistent parameter values but using participants’ best fitting parameter values for each model-independent choice), and found very similar results, now shown in supplemental (S2 Fig).

**S2 Fig.** **Participants’ best fitting parameters for model predicted choice.** Choice data was generated using each of the models using participants’ best fitting parameters, with 25 iterations. Those model-generated probabilities were then used to predict participants’ actual choices using equation 2.

One can see that the general logic maintains where the interaction coefficient of disjoint*eligibility is still not significant, suggesting that eligibility predicts choice equally well across conditions. More importantly, the disjoint*tabular interaction coefficient is still positive (indicated higher predictive accuracy of tabular is disjoint than conjoint) and significantly different from the disjoint*eligibility interaction (as no overlap in the confidence intervals). Thus, we conclude that either method appears to result in similar signatures, we have added a small sentence in the methods to clarify this - “In our second posterior predictive check, we also utilized the 25-iteration dataset, which uses participants’ best-fitting parameters to generate data, and found the same results as Fig 4.D (S2 Fig).” (P.30, L.661)

**2. Some panels in Fig. 3 are not referenced in the main text and could be moved to the supplementary materials to highlight the main behavioral signature of interest. While the effect of reward on stay probability in each combination of condition and time is the quantity of focus here, it is not directly visible from the estimated marginal means. It would be ideal if this effect could be summarized with a single number for each condition and time, maybe by treating reward as a single continuous predictor and looking at the regression coefficient under different conditions and trial times.**

We are in general agreement with the comment and wish to illustrate better to the reader where to direct their focal points. Thus, we have made two modifications to the panels shown in Fig 3. First, we have eliminated the conditions that generally are not important for our modes of comparison, so that Reward “=” and Time “1F” were removed, which allows one to focus on the comparison of positive vs negative rewards as well as immediate vs 2-trial forward outcomes. Second, we added additional text to indicate when the participants’ predicted probability overlaps with eligibility [E,-], tabular [-,T], both [E,T], or neither [-,-]. Additionally, we have reflected these changes in the figure, expanded the legend, and added additional interpretation. We have added these below from the manuscript.

**Fig 3. Behavioral signatures of learning and model predictions.** (A) This diagram demonstrates the logical structure of a delayed choice that reappears after three trials, categorized as either 'stay' (S) or 'switch' (W). It includes feedback valence for immediate and two-trials forward choices. Correctly utilizing two-trials forward information (illustrated with a curved arrow) suggests staying with the initial choice. (B) The probability of maintaining (i.e. staying on) a delayed choice three trials later were modeled using multilevel logistic regression, as a function of condition (Conjoint vs Disjoint), time (Immediate, 0F or two-trial forward, 2F), and reward (positive, + or negative, -), as well as their interactions (equation 1). This regression was run on data from participants (red), as well as data generated by the tabular model (green) and data generated by the eligibility model (blue) for comparison. The logistic regression’s estimated marginal means are displayed, accompanied by 95% confidence intervals for each condition. Text displays when participants’ estimated confidence intervals overlap with eligibility [E,-], tabular [-,T], or both [E,T]. (C-E) Collapsed two-way interactions for each of the combinations between the three variables, specifically time*reward collapsed across condition (C), condition*time collapsed across reward (D), and reward*condition collapsed across time (E). Bars represent 95% confidence intervals.

Given these new indicators, we can easily see when one model picks up on a pattern in participant data that the other model had not found and have now made this clear in the results.

Results, (P.11, L.241): “The three-way interaction shows that for the disjoint condition, the tabular model generally maps better than the eligibility trace, whereas, in the conjoint condition we have a mixture between the models. The two-way interactions help to identify the dimensions that are most prevalent for identifying differences in participant choice between the two models. For positive prediction errors, we can see that tabular tracks participants' tendency to switch as the eligibility and tabular models produced mixed results in negative prediction errors. Other effects are present but are a bit harder to cleanly map to one model or the other. Although these effects were significant, we were more concerned about how well the model tracked win-stay, lose-shift behavior in the participants. As such, visualizations of the interactions show that the tabular model followed participant behavior in the disjoint condition (Fig 3B-Bottom panels); whereas, in the conjoint condition eligibility and tabular had mixed patterns with participants (Fig 3B-Top panels). Collapsing on each dimension, we found that positive reward showed the win-stay and lose-shift heuristic (Fig 3C), which was predominately captured in the tabular model. Finally, collapsing on time we found that tabular picked up positive reward effects and mixed model effects when the reward effect was negative. All together, we find evidence for both models picking up unique patterns that might justify the use of a hybrid model.”

**3. Looking at the example pairs in Fig 4 is a solid approach but does not lead to a lot of additional insights. It is not surprising that both participants and the model could learn to change their behavior when reward contingencies change. Can this be used as evidence for or against either type of models?**

In order to provide more insights than just visualizing a few examples, we have conducted a more systematic analysis including all the pairs of stimuli used in the task. Below we present a table that illustrates the percentage that the model-predicted choice matched that of the participants’ actual choice per each pair. We also provide aggregate information on all pairs and pair-types below the table (Imm=Immediate, Del=Delay, and the number in parentheses represent the starting reward value of that stimulus).

| Pairs | Eligibility | | Tabular | | Hybrid | |
| --- | --- | --- | --- | --- | --- | --- |
|  | Disjoint | Conjoint | Disjoint | Conjoint | Disjoint | Conjoint |
| Imm(4) v. Imm(1) | 66.7% | 69.2% | 65.9% | 63.5% | 71.8% | 72.8% |
| Imm(4) v. Imm(-1) | 69.4% | 72.0% | 69.0% | 65.8% | 74.9% | 75.5% |
| Imm(4) v. Imm(-4) | 69.8% | 71.8% | 68.3% | 65.4% | 75.1% | 75.2% |
| Imm(4) v. Del(4) | 55.2% | 59.6% | 53.6% | 48.8% | 58.6% | 59.3% |
| Imm(4) v. Del(1) | 59.3% | 60.6% | 58.8% | 54.4% | 64.8% | 62.9% |
| Imm(4) v. Del(-1) | 61.1% | 61.0% | 59.7% | 55.1% | 67.2% | 63.3% |
| Imm(4) v. Del(-4) | 68.0% | 68.5% | 67.4% | 65.4% | 73.4% | 73.4% |
| Imm(1) v. Imm(-1) | 57.4% | 57.4% | 58.3% | 56.1% | 61.4% | 58.7% |
| Imm(1) v. Imm(-4) | 56.3% | 55.3% | 58.1% | 54.3% | 59.9% | 57.2% |
| Imm(1) v. Del(4) | 64.3% | 58.4% | 67.6% | 59.8% | 71.3% | 62.2% |
| Imm(1) v. Del(1) | 63.2% | 59.0% | 65.1% | 58.9% | 68.1% | 62.2% |
| Imm(1) v. Del(-1) | 61.4% | 60.3% | 62.2% | 59.6% | 65.5% | 63.5% |
| Imm(1) v. Del(-4) | 53.3% | 54.5% | 53.0% | 52.1% | 54.7% | 54.8% |
| Imm(-1) v. Imm(-4) | 55.8% | 56.2% | 57.0% | 55.4% | 59.1% | 58.1% |
| Imm(-1) v. Del(4) | 65.4% | 61.9% | 68.7% | 63.6% | 72.7% | 67% |
| Imm(-1) v. Del(1) | 62.6% | 61.7% | 64.9% | 61.8% | 68.6% | 66.1% |
| Imm(-1) v. Del(-1) | 59.6% | 60.8% | 60.6% | 60.6% | 63.1% | 64.1% |
| Imm(-1) v. Del(-4) | 57.3% | 58.0% | 58.2% | 56.1% | 61.9% | 60.0% |
| Imm(-4) v. Del(4) | 64.9% | 60.5% | 67.1% | 62.1% | 71.5% | 65.2% |
| Imm(-4) v. Del(1) | 63.3% | 59.1% | 64.8% | 59.2% | 69.8% | 62.8% |
| Imm(-4) v. Del(-1) | 61.1% | 59.2% | 61.4% | 58.0% | 65.6% | 62.2% |
| Imm(-4) v. Del(-4) | 53.8% | 53.8% | 55.2% | 52.1% | 57.1% | 54.7% |
| Del(4) v. Del(1) | 52.6% | 52.1% | 56.3% | 54.0% | 58.2% | 53.8% |
| Del(4) v. Del(-1) | 53.8% | 52.4% | 58.4% | 53.3% | 60.0% | 53.8% |
| Del(4) v. Del(-4) | 63.2% | 57.5% | 69.4% | 59.8% | 72.8% | 61.4% |
| Del(1) v. Del(-1) | 51.5% | 50.8% | 54.5% | 50.9% | 55.8% | 51.6% |
| Del(1) v. Del(-4) | 60.9% | 56.9% | 65.2% | 59.0% | 68.1% | 61.6% |
| Del(-1) v. Del(-4) | 58.4% | 56.4% | 61.2% | 58.8% | 63.5% | 61.0% |
| Mean | 60.3% | 59.5% | 61.8% | 58% | 65.5% | 62.3% |

First, we see that across all pairs, the eligibility generally maps better with participant choices in the conjoint condition (Eligibility = 59.5%, Tabular = 58%), whereas the tabular model maps better in the disjoint condition (Eligibility = 60.3%, Tabular 61.8%). However, the hybrid model was better mapped to participant behavior in both conditions (Conjoint = 62.3%, Disjoint = 65.5%).

Second, Eligibility did better in immediate vs. immediate pairs (Eligibility = 63.1%, Tabular = 61.4%), Tabular did better in delayed vs delayed pairs (Eligibility = 55.5%, Tabular = 58.4%), and both models were about equivalent in mixed types (Eligibility = 60.3%, Tabular = 59.9%). Similar to the condition effects above, the hybrid model performed better in all three types of pairs than the two independent models (immediate = 66.6%, delayed = 60.1%, mixed = 64.3%).

We have now added a new panel to Fig 4 (Fig 4.C) and edited the legend to reflect on this (P.15, L. 324).

**Fig 4 Model Prediction of trial-by-trial choice.** … (C) Predictive accuracy (i.e. percentage of model-predicted choice matching participant choice) for each model and each pair type with delayed vs. delayed (Del v. Del), immediate vs. immediate (Imm v. Imm), and immediate vs. delayed (Mixed), shown as a boxplot across participants. …

One can see that all models perform best in immediate vs. immediate pairs and worst in delay vs. delay pairs. The hybrid model performs better than single-strategy models in all three pair types, where this advantage is highest in the mixed pair type. We also added a few sentences in our results (P.13, L.291): “Next, we set out to see if our models behaved systematically different across the different pair types, and utilized our previous 25-iteration simulated dataset. For each of the three models we calculated the averaged model accuracy (i.e., how well the model predicted participants' choices) separately for each pair type. We show that tabular appears to perform better than eligibility in delay vs. delay pairs, and worse than eligibility for immediate vs. immediate pairs (Fig 4C). The hybrid model performed better for all pair types (Fig 4C, red bars), suggesting that given its six parameters, it may find parametric spaces that accurately map immediate, delayed, and mixed pair types without overt bias towards one or the other.”

**4. In Fig 4C, the fixed effect of disjoint * eligibility is not significant, contradicting the figure caption.**
We apologize for the oversight, we have corrected this error (P.14, L.320), “For the interaction between eligibility predictions and condition, we found a numeric reduction in the odds-ratio, but this effect was not significant, b = .92, 𝛸^2^(1, 26) = 2.45, p = .12.”

**5. In the conjoint condition, do the estimated values used to calculate prediction error (Eq. 4 and 7) correspond to the sums of the current action and action two trials back? This seems more sensible, since participants should know that the reward is the sum of immediate and delayed rewards. The equations seem to suggest that the prediction error is calculated only using the current action (Eq. 4) or separately for the current and previous action (Eq. 7).**

We apologize for the ambiguity and would like to further clarify how expected values and prediction errors are calculated for each condition and model.

The eligibility trace model only contains a single estimated expected value for each of the options. Thus, these value estimates reflect the options that were deemed eligible to receive discounted updates of the current trial prediction error. This prediction error is the difference between the summed (immediate + delay) reward feedback and the current selection’s estimated chosen value. There is no difference in update between the two experimental conditions. This clarification has now been added in the Methods section (P.28, L.599): “This algorithmic implementation is performed in both conjoint and disjoint conditions.” We implemented this method primarily due to its generalizability (Chapter 12, Sutton & Barto, 2017) to a broad range of environments. The prediction error is only used to update the value of the option selected on the current trial; therefore, it should rely on the estimated value of that action only. The 2-trial back action is updated through the trace going back in time, rather than ‘model-based’ knowledge of incorporating the 2-trial back option.

The tabular model performs a different type of update between the two conditions. In both conditions, two prediction errors are calculated: one used to update the current selection value, and one used to update the 2-trial back selection value. We have now clarified the difference between the two conditions in the tabular model description section in the Methods (P.29,L.628). “In the conjoint condition, the first prediction error is calculated as the summed reward minus the current selection's value estimate, while the second prediction error is the difference between the same summed reward and the value estimate of the delayed selection from two trials back. This is akin to an eligibility trace with only updating the two signal states (T and T-2). In the disjoint condition, however, the tabular model makes use of the full explicit feedback and performs the same sort of double update. This double update uses the dissociated reward function to calculate the two prediction errors, where the immediate reward is compared to the current selection and the delayed reward is compared to the selection two trials back.” Along with this clarification, we made slight updates to our prediction error equation (Eq. 7).

**6. In the disjoint condition, it seem plausible that tabular-based learners (or maybe even eligibility trace-based learners) would calculate two separate prediction errors using the immediate and delayed rewards, which doesn’t seem to be what Equation 7 suggest. How is this taken into account by the models?**

The tabular model does indeed calculate separate prediction errors, like the reviewer suggests. As mentioned in the response to the previous comment, we have now clarified this in the Methods section describing the tabular model. The eligibility trace model in its current implementation does not take this into account.

**7. Is z-scoring the values crucial for the results? It seems like an unconventional choice. How would the results change without it?**

We thank the reviewer for allowing us to clarify the reasons why we z-scored values during action selection. The main purpose was to allow valid comparisons across our two conditions given the nature of the three random walks and each individual’s random presentation of choice pairs. Most experimental reinforcement learning tasks utilize a yes (1) or no (0) reward function with some incorporating punishment (-1) but the majority of tasks focus on fixed magnitudes and reversal of probabilities rather than reversals of reward. To handle the variation in our differing random walks, we implement a rolling average of the last 5 trials that normalizes the vector of mean-adjusted value estimates using the standard normal distribution. Otherwise, the parameters might contain weighting problems (such as a small parameter for large values or a large parameter for small values), when placed for statistical analysis. To make this more explicit, our random walks are not restricted to any max or min reward, and will sometimes randomly walk outside of the initial boundaries of +4 and -4 (see y-axis below). This would end up weighing some of the inverse temperatures based purely on the random walk a participant is in and make the random walks not comparable. Additionally, the experimental conditions will also make differences in our temperatures, due to the summation of reward to produce higher degrees of positive or negative prediction errors (such as an immediate 4 + delayed 4 to produce a summed 8).

Given these three random walks, larger value differences will lead to systematic reduction in our inverse temperature estimates. This might be akin to a regression equation where if you scale your data, then your coefficients will change as well. For example random walks 2 and 3 appear to have higher inverse temperature parameters to complement their smaller reward differences. We also supply a table to show the mean changes in our strategy weight parameter values and participants number (n) per the random walks and scaling.

|  | Condition | Beta Elg | | Beta Tab | | n |
| --- | --- | --- | --- | --- | --- | --- |
|  |  | No scaling | Rolling z-sc | No scaling | Rolling z-sc |  |
| Random Walk 1 | Conjoint | .86 | .67 | .67 | .38 | 47 |
|  | Disjoint | .68 | .75 | .75 | .57 | 55 |
| Random Walk 2 | Conjoint | 1.3 | .87 | .87 | .46 | 46 |
|  | Disjoint | 1.6 | .72 | .72 | .59 | 50 |
| Random Walk 3 | Conjoint | 2 | 1.39 | 1.4 | .5 | 49 |
|  | Disjoint | 1.14 | .85 | .85 | .53 | 37 |

You may notice that the biggest difference comparing the conditions (n_conjoint_ – n_disjoint_) sample sizes exist for random walk 3 (+12) as compared to random walk 2 (-4) or random walk 1 (-8). This systematic bias produces greater values for the conjoint condition, where our standardization weakens the effect. Thus, despite this systematic bias, we still produce significant effects in the opposing direction for our two experimental conditions.

We have modified a sentence in our main text to further clarify why the values were z-scored (P.30, L.648), “By normalizing the data before feeding into the SoftMax, the individual and hybrid models can account for different scales of the random walks and reduce the noise of sub-models operating on different scaled rewards.”

**8. The model comparison results could be corroborated with more careful analyses and interpretation. In Table 2, having AIC, BIC, and pseudo-R^2 for model comparison feels redundant. When comparing the hybrid model with the two other models, pseudo-R^2 is not very useful since it does not take into account parameter count. Furthermore, the results of AIC and BIC contradict each other in three of the four conditions, which makes the result inconclusive. The authors may wish to perform model recovery using the two metrics to identify which metric leads to better recovery of the true model. The comparisons between eligibility and tabular models are not affected as much since they have the same number of parameters, but it’s hard to tell the effect size of their differences without knowing the standard error of these metrics. Bayesian model selection could also be useful.**

We appreciate the suggestion from the reviewer and have used the 25-iteration model choice dataset generated with the best-fitting model parameters then refitted by each model to calculate the AIC and BIC for each participant, condition, and iteration. Below we present a table of the percentage of simulated datasets for which the generating model was correctly recovered (i.e. also fit the simulated data best). We also placed this table into our supplemental materials (S2 Table).

**S2 Table. Model Recovery.**

| **AIC** Recovery Rate | Disjoint | Conjoint |
| --- | --- | --- |
| Eligibility | 96.1% | 96.5% |
| Tabular | 96.8% | 96.6% |
| Hybrid | 82.8% | 79.8% |
| **BIC** Recovery Rate | Disjoint | Conjoint |
| Eligibility | 96.1% | 96.5% |
| Tabular | 96.8% | 96.6% |
| Hybrid | 69.1% | 60.2% |

We generated 25 iterations of model generated choice and then refitted them with each model. Percentages show how often each model comparison metric (Akaike information criterion, AIC and Bayesian information criterion, BIC) recovers the true generated model. Due to the better recovery on hybrid, we chose to continue model comparison using AIC.

Additionally, pseudo-R^2^ was reported more as an intuition for the reader to understand the proportion of variance accounted for, than as a means of model comparison. We have therefore left pseudo-R^2^ and AIC in Table 2, but with the extra clarification in the table legend that pseudo-R^2^ was not used for model comparison. (P.16, L.364): “Pseudo-R^2^ represents the proportion of variance accounted for in participants' choices relative to a random model with higher values representing better fit. Pseudo-R^2^ was not used for model comparison and is provided for general intuition of model accounted variance.”

**9. Based on Table 2, conjoint conditions are better fit by eligibility models, but disjoint conditions (especially stage 2) are not necessarily better fit by tabular models. This contradicts the heading of the previous section, and seemingly also the result in Fig. 4C. Will the results change if the models were simulated using participants’ best-fitting parameters?**

Given the reviewer’s advice and our additional analysis, we believe that the changes we implemented should augment our rationale for our final interpretation and are now consistent between Table 2 and Fig 4D (former Fig 4C). We provide the changes to Table 2 as follows (P.15, L.346).

“After validating our models, we proceeded to fit them to the data and extract model-fitting metrics and parameter values (see Methods for details about the model-fitting procedure). We provided two descriptive statistics of model fit averaged across participants for each condition (Table 2): Akaike Information Criterion (AIC) and pseudo-R^2^. Across all conditions and stages, our hybrid model accounted for the most explained variance, but the additional parameters did not always merit a better fit depending on both condition and stage (Table 2). In most cases, the hybrid model shows a better fit to participant data except in Conjoint-1, where the hybrid model cannot be differentiated from the eligibility model (Δ AIC < 2). To note, the hybrid model can be reduced to either the eligibility or tabular model but will still be penalized for the additional parameters. Tabular only appeared to fit better in the Disjoint-1 condition as compared to eligibility in all other conditions. Consistent with our posterior predictive checks from Fig 4D, the eligibility model fit did not differ across conditions, while the tabular model fit improved in the disjoint condition (compared to conjoint). Participants who started in the disjoint condition had better model fits than those who started in the conjoint condition, which aligns with our previous statistics (Table 1).”

**Table 2. Average Model-Fitting Metrics.**

|  |  | AIC | | | Pseudo-R2 | | |
| --- | --- | --- | --- | --- | --- | --- | --- |
|  |  | Eligibility | Tabular | Hybrid | Eligibility | Tabular | Hybrid |
| Group D->C (N=74) | Disjoint-1 | 360 | 357.5 | 342.5 | .24 | .245 | .29 |
|  | Conjoint-2 | 360.9 | 383.2 | 358.3 | .24 | .19 | .26 |
| Group C->D (N=68) | Conjoint-1 | 391.5 | 410.9 | 391.3 | .17 | .13 | .19 |
|  | Disjoint-2 | 391 | 400.2 | 383.7 | .17 | .15 | .2 |

Average model-fitting metric (Akaike information criterion, AIC) for each model across condition and stage, with lower AIC values representing better fit. Pseudo-R^2^ represents the proportion of variance accounted for in participants' choices relative to a random model with higher values representing better fit. Pseudo-R^2^ was not used for model comparison and is provided for general intuition of model accounted variance.

**Reviewer #2: Maulhardt et al. investigated how humans engage in temporal credit assignment. Using a modified two-armed bandit task, the authors have shown that humans uses a hybrid of eligibility trace and tabular update, the weight of which is influenced by the degree of information uncertainty. The authors have used a variety of analyses to demonstrate that participants are able to perform this temporal credit assignment problem and that the posterior predictive check of different models matches participants behavior under joint/disjoint conditions.**

**1. Despite the importance of the research topic and solid data analysis, I am not sure if the current set of experiments and analysis convinces me that the eligibility trace and tabular update models are the most suitable model candidates to account for human’s decision making in this temporal credit assignment task. This is mainly because the authors have not tested other competing models (e.g., single step TD learning) or reduced version of the two main models (e.g., fix some parameters to a fixed value) to justify that all the parameters are necessary. The overall inconclusive results on the model comparison (AIC, BIC) also makes me wonder whether splitting people based on their individual’s best fit models and examining their behavior could provide more insights into how the usage of different algorithms is related to different behavioral patterns.**

We appreciate the reviewer’s input and have now implemented their suggestions in three ways:

First, we have tested six additional models to further justify our proposed eligibility and tabular mechanisms and validate their parameters (S1 Table).

Second, after taking into account Reviewer 1’s suggestion regarding model comparison metrics, we now focus on AIC given its stronger ability to accurately recover the true model in a model recovery analysis (compared to BIC). This has helped make the results more consistent throughout the manuscript.

**S1 Table. Additional Model Comparisons.**

| AIC | Group: Disjoint → Conjoint (N=74) | | Group: Conjoint → Disjoint (N=68) | |
| --- | --- | --- | --- | --- |
|  | Disjoint-1 | Conjoint-2 | Conjoint-1 | Disjoint-2 |
| Eligibility | 360 | 360.9 | **391.5** | 391 |
| Eligibility-Fix | 380.2 | 387.3 | 414.8 | 411.8 |
| Tabular | 357.5 | 383.2 | 410.9 | 400.2 |
| Tabular-Fix | 368 | 396.6 | 421.2 | 410.7 |
| Hybrid | **342.5** | **358.3** | **391.3** | **383.7** |
| Hybrid-1L | **343.8** | **359.3** | **391.4** | **384.2** |
| Hybrid-1L1D | **343.7** | **358** | **390.1** | **383.3** |
| Hybrid-Fix | 354.9 | 379.6 | 408.7 | 398.4 |

Six additional models were tested in relation to our hybrid model using Akaike information criterion (AIC) as our model comparison metric: (i) the classic temporal difference learning algorithm (TD) which is akin to updating the current action and one-step back (rather than two-steps like in tabular); (ii) an eligibility-tabular hybrid with a shared learning rate (Hybrid-1L) between both models; (iii) an eligibility-tabular hybrid with a shared learning rate and shared decay rate (Hybrid-1L1D) between both models; (iv) an eligibility model with fixed learning and decay rates (Eligibility-Fix) using the mean best fitting parameters from the eligibility model; (v) a tabular model with fixed learning and decay rates (Tabular-Fix) using the mean best fitting parameters from the tabular model; and (vi) a fixed hybrid model (Hybrid-Fix), using the mean best fitting parameters from the hybrid model. We bolded the best fitting models (Δ AIC < 2) for each condition and selected the full model for analysis (to maximize explanation). However, most shared parameter models (1L and 1L1D) showed similar results to the full hybrid model.

Originally, we had planned to perform analysis with the 1L model but figured to focus on greater explanatory power (two learning rates for each strategy), but the results were rather similar (as indicated with the Δ AIC < 2). As such, we continued analysis using the 6-parameter hybrid model and hope this table reinforces the use of the hybrid model to widen the parameter space to participants’ strategies. It is also interesting to note that the hybrid model appears to reduce down to the eligibility model in the Conjoint-2 condition, where if selecting for the most parsimonious model (to maximize prediction) would suggest selection of eligibility over hybrid.

Third, we appreciate the reviewer’s suggestion of splitting people into groups based on their individual best-fitting models; however, our sample was not powered for such separation into smaller groups. We have added a sentence to the discussion to mention exploring this variability in future work: “Given more power, one could consider fitting each individual to their best fitting model to gain more insights, rather than relying on a one-fits-all model.” (P.24, L.506).

**2. I am also not totally convinced by the argument of temporal uncertainty influences the strategy adopted during temporal credit assignment. Why it is not just task difficulty? If the argument is about task difficulty, let’s think about another hypothetical experiment where delayed reward is 3 steps into the future. Since the task is harder (requires to hold more distant items in working memory), I would expect that participants put more weight on the eligibility trace algorithm, while in this case the information uncertainty I assume is the same as 2-step situation since the immediate and delayed reward is always separated. I would suggest the authors either conduct more experiments that manipulate information uncertainty in different ways to provide more support for this argument, or maybe be more careful and clearer when defining what they mean by uncertainty.**

We certainly agree with the reviewer that increased uncertainty (whether temporal or information uncertainty) would generally be associated with increased task difficulty, regardless of the task, and this is not a trivial confound to address.

First, we would like to clarify the difference between temporal and information uncertainty and emphasize that our findings are consistent with information uncertainty (and not temporal uncertainty) influencing the strategy adopted during the task, since our task design explicitly manipulated information uncertainty through the conjoint and disjoint condition, while keeping temporal uncertainty constant across conditions. It is indeed possible that temporal uncertainty would also influence the weight participants put on eligibility trace vs tabular, but this question was outside the scope of the current study. In our view, temporal uncertainty is inherent in delayed outcomes, but may affect more than just difficulty (Bennett et al., 2016; Kobayashi & Schultz, 2008). We have reviewed the manuscript and changed any claims about temporal uncertainty influencing strategy use. We have also modified the title to reflect the type of manipulation we have performed: “Information uncertainty influences learning strategy from sequentially delayed rewards.”

Difficulty, expectation, discounting, and credit assignment are also potential downstream results of temporally extending the inherent uncertainty of delay. Therefore, while the manipulation suggested by the reviewer (of extending the delay to 3 or more trials, while maintaining information uncertainty constant) would allow establishing that information uncertainty is not the only factor that influences strategy use, it would not necessarily separate the contribution of task difficulty from temporal and/or information uncertainty. Although increasing the number of delay steps might produce more task difficulty, there may also be unintended trade-offs of value and time such as with discounting. Thus, it might be best to utilize this task with varying delays of different stimuli. Then we might be able to calculate the individual tradeoffs for value and time for our two algorithmic solutions. One might also consider implementing some of the manipulations by Gershman et al. (2014), which modulated working memory capacity with secondary tasks, increased trials, and a resting period. Their purpose was to find if experience replay would show more model-based behavior but still resulted in multiple alternative interpretations. As such, the temporal credit assignment problem carries properties that are difficult to delineate in a clean fashion. However, some of our future studies might look more towards the balance between value and time using either of these previous methods.

In addition to our points above, we have done additional analysis and added a task difficulty figure in our supplementary material (S1 Fig). Although we are not able to fully tease apart the effects of task difficulty and information uncertainty, we believe this additional analysis illuminates how the two models differentially handle task difficulty and information uncertainty.

**S1 Fig. Task Difficulty.** (A). Perceived task difficulty ratings collected at the end of the study were not significantly different between our two groups of participants (those who started with the conjoint condition vs those who started with the disjoint condition, t(140) = -0.12, p = .9). (B). Using a linear regression with an interaction between perceived difficulty and dummy code for decision weight on AIC showed two main effects (main effect of perceived difficulty, t(565) = -2.13, p = .03, and main effect of strategy t(565) = -10.17, p < .001) but no interaction effect, t(564) = .74, p = .46. This suggests that perceived task difficulty might just make people more stochastic and less consistent in their choices, rather than more likely to rely on eligibility traces. (C). AIC values for each of the eligibility and tabular models with average rate of feedback change per participant. The feedback rate was calculated as 0 when the selected object’s reward value did not change from the last time it was previously selected and 1 otherwise, with the assumption that higher feedback change rate is associated with higher task difficulty. We fit two linear regressions predicting the AIC of eligibility and the AIC of tabular with two predictors of condition and feedback change rate. For both models we found a significant main effect of feedback change rate, for eligibility: t(278) = 3.68, p < .001, for tabular: t(278) = 4.89, p < .001. However, there was no effect of the condition for eligibility: p = .48, but there was an effect for tabular: t(278) = -3.12, p = .002. Thus, there appears to be a linear effect of task difficulty and model fit but our reward manipulation only appears to uniquely contribute to our tabular model.

We added a short sentence in our methods and modified our discussion to reflect these considerations about task difficulty:

Methods (P.26,L.566): “We also conducted additional analyses to assess whether task difficulty was a contributing factor to our experimental conditions and model-parameter weights (S1 Fig).”

Discussion (P.24,L.504): “Another possibility for condition and/or order effects could lie in the difficulty of the task itself being different between conditions (i.e. harder in conjoint, under low information uncertainty). Given more power, one could consider fitting each individual to their best fitting model to gain more insights, rather than relying on a one-fits-all model. While we were unable to fully isolate the effects of task difficulty from information uncertainty in our design, our supplemental analyses (S1 Fig) suggest that our findings hold when controlling for task difficulty. Future experiments may additionally consider using variable delays to help separate the role of information uncertainty, temporal uncertainty and task difficulty. However, capturing a single interpretation in RL strategy is oftentimes confounded when considering the vastness of human cognition^12,36^. We should be mindful of the greater range of possible solutions when learning under temporal uncertainty and future studies might look into modifying these models for preservation, working memory, or learning rate asymmetry^37–39^. Preservation appears to be a vital strategy in either reducing or eliminating the credit assignment problem and maybe adaptive when presented with long-term temporal uncertainty^40,41^.”

**3. The authors should cite Bruinsma, S., Petzschner, F., & Nassar, M. Perseverative Behavioral Sequences Aid Long-Term Credit Assignment. (A CCN proceeding)**

This looks right on topic, we appreciate the recommendation and have now added the citation to our Discussion (P.24, L.515): “Preservation appears to be a vital strategy in either reducing or eliminating the credit assignment problem and maybe adaptive when presented with long-term temporal uncertainty^40,41^.”

**4. On Figure2, I assume O1 means Observation 1? It can be easily mistaken to be 0. I would recommend either write out a longer abbreviation (obs?) or make it more clearer about what S and O stands for in the caption.**

O1 actually stands for object 1 in this case, we have modified the figure and the legend to be more clear (Changed O1 to Obj1).

**5. On page 10, the authors said that ‘we selected sequences where participants chose a delayed option and it reappeared as a potential choice three trials later’ - it would be helpful to know how many trials (the proportion of the total trials) this subset refer to.**

We have now added this information to the Results section when we describe this analysis (P.11, L.227): “Overall, about 15.7% of the original dataset was subset and across individuals showed a range of 10.5% to 22.8%.”

**6. On page 11, I am not sure I understand the interpretation of the three-way interaction disjoint*+*2F. What is the baseline level that this interaction term is compared to? To get a better idea of what a three way interaction is, it may be helpful to split the data based on one of the factor and redo two-way interaction to examine the effect more directly.**

Given some of the concerns from Reviewer #1, we have modified this analysis to remove conditions that weren’t helping distinguish between participant behavior and our two models, specifically the neutral feedback and the 1-trial forward (1F) time. The baseline level is conjoint*-*0F; however, the significance of the difference coefficient (disjoint*+*2F) justifies the use of a three-way interaction. Although one could break this down more into more regression models, we have chosen the more parsimonious and statistically valid method of fitting the overall model and then collapsing on dimensions with the ‘emmeans’ package. Additionally, we wished to see the three-way interaction in relation to the model-predicted choice. Thus, we have chosen to use a graphical representation to further explore the relationship that was identified in our three-way interaction. We have updated our analysis, interpretations, and figure below (P.11, L.232).

“Specifically, the regression examined whether the experimental conditions (*Condition: conjoint, disjoint*) influenced participants’ decision to stay (*Stay: 1 stay, 0 switch)* while accounting for the reward valence (*Reward: + positive feedback, - negative feedback*) received from current and two-trials forward (*Time: 0F, 2F*). For example, a participant might erroneously stay on their choice three trials in the future due to a positive immediate reward but should have correctly switched due to a negative reward two-trials in the future (Fig 3A). Using equation 1, we conducted three logistic regression analyses: one with the participants' data, and two with a simulated dataset using 25 iterations of each participant’s best-fitting parameters (see Methods for details) for both the eligibility and tabular choice models (Fig 3B).

The three-way interaction (disjoint*+*2F) was significant for all three logistic regression models (Fig 3C), odds-ratios for participant, *b* = 2.34, SE =1.16, *Z* = 5.88, *p* < .001, 95% CI [1.77, 3.13]; eligibility, *b* = 1.16, SE =1.07, *Z* = 2.22, *p* = .03, 95% CI [1.02, 1.32]; tabular, *b* = 2.38, SE =1.11, *Z* = 8.48, *p* < .001, 95% CI [1.95, 2.9]. For participants’ data (Fig 3B – red line), the interaction was such that in the conjoint condition, the reward valence on choice (probability of stay difference between positive versus negative rewards) was strongest for immediate (0F). However, in the disjoint condition, the effect of reward valence on choice was strongest for two-trials forward (2F). The three-way interaction shows that for the disjoint condition, the tabular model generally maps better than the eligibility trace, whereas, in the conjoint condition we have a mixture between the models. The two-way interactions help to identify the dimensions that are most prevalent for identifying differences in participant choice between the two models. For positive prediction errors, we can see that tabular tracks participants' tendency to switch as the eligibility and tabular models produced mixed results in negative prediction errors. Other effects are present but are a bit harder to cleanly map to one model or the other. Although these effects were significant, we were more concerned about how well the model tracked win-stay, lose-shift behavior in the participants. As such, visualizations of the interactions show that the tabular model followed participant behavior in the disjoint condition (Fig 3B-Bottom panels); whereas, in the conjoint condition eligibility and tabular had mixed patterns with participants (Fig 3B-Top panels). Collapsing on each dimension, we found that positive reward showed the win-stay and lose-shift heuristic (Fig 3C), which was predominately captured in the tabular model. Finally, collapsing on time we found that tabular picked up positive reward effects and mixed model effects when the reward effect was negative. All together, we find evidence for both models picking up unique patterns that might justify the use of a hybrid model.”

**Fig 3. Behavioral signatures of learning and model predictions.** (A) This diagram demonstrates the logical structure of a delayed choice that reappears after three trials, categorized as either 'stay' (S) or 'switch' (W). It includes feedback valence for immediate and two-trials forward choices. Correctly utilizing two-trials forward information (illustrated with a curved arrow) suggests staying with the initial choice. (B) The probability of maintaining (i.e. staying on) a delayed choice three trials later were modeled using multilevel logistic regression, as a function of condition (Conjoint vs Disjoint), time (Immediate, 0F or two-trial forward, 2F), and reward (positive, + or negative, -), as well as their interactions (equation 1). This regression was run on data from participants (red), as well as data generated by the tabular model (green) and data generated by the eligibility model (blue) for comparison. The logistic regression’s estimated marginal means are displayed, accompanied by 95% confidence intervals for each condition. Text displays when participants’ estimated confidence intervals overlap with eligibility (E,-), tabular (-,T), or both (E,T). (C-E) Collapsed two-way interactions for each of the combinations between the three variables, specifically time*reward collapsed across condition (C), condition*time collapsed across reward (D), and reward*condition collapsed across time (E). Bars represent 95% confidence intervals.

**7. On page 13, the authors claimed that their priorr parameter selection ‘maintained consistent parameters across the two models’ - how is this achieved?**

We wanted to ensure that the model differences were due to algorithmic differences rather than differences in parameter value input. Such that, the learning rate, decay rate, and inverse temperature parameter input for eligibility and tabular were the exact same numeric value. We detail this in our results, “To test this, we employed the same task sequences observed by participants and utilized a beta distribution (α = 1, β = 2) for our learning rate, a gamma distribution (α = 3, β = 1) for the inverse temperature parameter, and a beta distribution (α = 2, β = 3) for the decay parameter. This maintained consistent parameters across the two models and kept a high inverse temperature parameter to reduce random noise.” (P.14, L.310)

**8. For Figure4, it seems that for all the pairs, the initial better option will in the end drift away to be the inferior option - is this by design in the pseudo-random random walk schedule? I understand that the restless aspect of the task is to make sure that the participants keep learning, but why introduce this structural drift early vs. late task?**

The random walks were gaussian noise from their starting point (σ = .25), but in some cases there were obvious reversals that could illustrate some of the analysis given by Tanaka et al. (2007). We purposefully picked example pairs that show a clear reversal to better visualize learning behavior. However, there were also pairs with a lot of reversals, others with very few, or none. We have added clarification in the manuscript in Fig 4 legend (P. 15, L. 324), “(A) Average participants’ propensity to choose left on three example pairs from one of the reward random walks, selected to show a clean reversal moving from left-to-right choice.” Because there is a total of 3 random walks * 28 pairs, we have chosen another subset to show the reviewer. Overall, this created enough variability between the pairs, and we did not explicitly control when in the task structural drift occurred.

**9. What is the overall distribution histogram of the weight parameter in the hybrid model?**

We plot a histogram below showing the distribution of the two weight parameters from the hybrid model (along with learning rate and decay rate). For completeness we have now added a supplementary figure (see S3 Fig) showing the parameter distributions from all three models.

**S3 Fig. Distribution of Model Parameters.** The distribution of learning rate (alpha), inverse temperature weight (beta), and decay rate (lambda) from eligibility (elg) and tabular (tab).

Thank you again for your time and consideration, we look forward to hearing from you.

Sincerely,

Caroline J. Charpentier, PhD	Sean R. Maulhardt, MA

Email: [ccharpen@umd.edu](mailto:ccharpen@umd.edu)	Email: [smaulhar@umd.edu](mailto:smaulhar@umd.edu)
