# Supplementary material for: Information uncertainty influences learning strategy from sequentially delayed rewards
Source: PLoS Comput Biol. 2026 Feb 2;22(2):e1013879. doi: 10.1371/journal.pcbi.1013879 (PMC12885371; doi:10.1371/journal.pcbi.1013879)
Supplement: Attachment — Submitted filename: Response to Reviewers_v2.docx [file pcbi.1013879.s016.docx]

Dear Reviewers,

Thank you for reviewing our revised manuscript, “*Information uncertainty influences learning strategy from sequentially delayed rewards*” (PCOMPBIOL-D-25-00171) and for your questions and concerns. We were glad to see the reviewers found our revised manuscript improved, and have addressed the remaining questions and concerns in the following ways.

Specifically, we have (1) further characterized the differences between our two computational models (new S2 Fig), (2) added analyses of the differences between non-scaling and scaling methods for our value function (new S3 Fig) and justification for the scaling, (3) used a signed-rank test for comparison between AIC model-fit metrics (Table 2 and S2 Table), and (4) provided greater detail for our model-fitting procedure. We have addressed both reviewers’ concerns with our 5-trial rolling z-score and hope they find the analysis to provides substantive justification. As pointed out by Reviewer #1, we attempted to give better insight into the qualitative predictions of our two models and used a signed-rank test for significance comparisons of model-fit. We also made many of our figures more robust to address concerns of Reviewer #2, along with providing more details for our model-fitting procedures.

For ease of reference, we have added your comments below in bold (with subpoints in bold-italics) followed by our responses with page and line number for any changes within the manuscript.

With these additional revisions, we hope the manuscript will be suitable for publication at *PLoS Computational Biology*.

Again, we appreciate the time and consideration from both reviewers, and we look forward to hearing from you.

Sincerely,

Caroline J. Charpentier, PhD	Sean R. Maulhardt, MA

Email: [ccharpen@umd.edu](mailto:ccharpen@umd.edu)	Email: [smaulhar@umd.edu](mailto:smaulhar@umd.edu)

**Reviewer #1**

**I appreciate the author’s thorough effort in the revision which has substantially improved the manuscript. I have a few follow-up comments:**

Thank you - we are happy to see that the reviewer finds the revised manuscript substantially improved and hope we have addressed the follow-up comments adequately.

**1. The authors have simplified and clarified Fig. 2, but I still find it somewhat hard to interpret. It would be great if we can see qualitatively distinct predictions made by the two models (similar to Fig. 1B in Witkowski et al., 2025, and Fig. 2E-F in Jocham et al., 2016), although the quantitative comparisons are certainly valid and consistent with the rest of the paper. I believe the eligibility model predicts that positive/negative outcomes at trial T lead to more/less likelihood to repeat choices made in trial T, T-1, T-2, T-3, etc. with decreasing effect. The tabular model predicts that reward outcomes at trial T gets attributed only to choices in trial T and T-2, and nothing else. This should be especially apparent if we look at the simulations, but it is hard to discern currently. I believe looking at the difference between P(stay|+ outcome) and P(stay|- outcome) would reflect win-stay lose-switch more clearly than just looking at the marginal means individually.**

The reviewer’s intuition about how the two models might make qualitatively distinct predictions is correct; however, in the specific experimental context of our task, this pattern will not show a smooth gradient due to the random presentation of pairs and the random walk of reward. That is, the T-1 outcome does not have any systematic relationship to the current choice and therefore, any outcome is equally possible for any of the possible choices. The eligibility model will gradually credit previously selected objects but over time the trace will approximate the true value function with noise from T-1 and T-3+ (given the lack of systematic relationship) and with appropriate credit to the choices made at T or T-2.

We did attempt a simulation similar to what the reviewer suggested; in which we generated choices from the two models (along with analyzing participants’ actual choices) and calculated the adherence to a win-stay lose-switch (WSLS) heuristic (as a proxy for outcome sensitivity) on the current choice, based on the previous three trials’ choices and outcomes. The following graph shows for the current trial, whether the participant chose an option that was chosen one-trial back (stay lag 0), two-trials back (stay lag 1), or three-trials back (stay lag 2); otherwise, if the option didn’t appear, then it was excluded from analysis. We also yoked this with the reward received on those lagged trials, such that if the participant performed a stay with a positive reward or a switch with a negative reward then they received a higher proportion of utilizing the win-stay, lose-switch heuristic (+1). However, if they did the opposite, such as switching on a positive reward or staying on a negative reward, then they received a lower proportion of the heuristic adherence (-1). This was done to aggregate the dimensions of information and make the graph more palatable for the reader.

The graph shows that both models are learning the signal, with eligibility appearing to perform better than tabular (i.e. closer to participants’ data) in the conjoint condition for objects with immediate rewards - this can be seen in the diagonal values of the first column, highlighted with red boxes, with WSLS adherence values around 0.19 for eligibility but 0.15 for tabular. Meanwhile, tabular appears to perform better in the disjoint condition for objects with delayed rewards - this can be seen in the bottom left (reward lag 0, stay lag 2) values of the right column, also highlighted with a red box, with WSLS adherence values of 0.301 for tabular but 0.237 for eligibility. While these patterns are consistent with our findings, this graph doesn’t necessarily draw strong qualitative or quantitative differences between the two models because both models are able to find the systematic association between objects and their immediate or delayed outcomes. Therefore, we did not add this analysis to the manuscript given that our analyses with the marginal means (current Fig 3 and associated results) allowed us to make more quantitative claims but would be happy to do so if the reviewer deems it necessary.

Rather, further simulation analyses have helped us identify that the difference between the models is primarily based upon their efficiency in handling the experimental presentation of rewards. To demonstrate this difference more clearly, we set up 3 participants’ experienced reward trajectories (one for each of the random walks) and their random presentation of pairs. We fixed their parameters and matched each reward-feedback condition to the same reward trajectory and presentation of pairs experienced by the participant. We ran this data through our models and plotted the change in value function over time (with the random walks) for each model.

**S2 Fig. Value function evolution over time.** This figure illustrates the evolution of value functions over time for three representative participants (one for each random walk, labelled 1, 2, and 3 in the facet title) during the presentation of the same experienced stimulus pair trajectory of each participant. The analysis compares the Eligibility Trace (Elg) and Tabular (Tab) learning models under identical conditions: fixed learning parameters (α = 0.2, β = 1, λ = 0.8) with both information uncertainty manipulations (conjoint vs. disjoint conditions). Each panel displays value changes throughout the experimental session, highlighting the distinct learning trajectories and convergence patterns between the two computational models. The tabular model shows smoother learning curves, especially in disjoint, while eligibility has more noise in the value function with more erratic changes and credit jumps.

These analysis and new figure indicate that eligibility has a non-broken credit trace to the recency of chosen objects and shows a large degree of noise in its value function due to the trace being inappropriately applied to randomly related stimulus-outcome pairs, and thus, the value function reflects this noise, resulting in more erratic changes in trial-by-trial value estimates seen in the graph above. The tabular model is able to overcome this noise and apply credit appropriately to the systematically related stimulus-outcome pairs, leveraging the information in the disjoint condition to a higher degree than eligibility, thus leading to overall smoother changes in the value function (especially in the disjoint condition).

We have now added this new figure to the supplemental materials, as well as a sentence to our methods section (p.30, l.666), “This double update uses the expanded reward function to calculate the two prediction errors, where the immediate reward is compared to the current selection and the delayed reward is compared to the selection two trials back. Given our experimental design, this leads to a smoother gradient of update for tabular compared to eligibility, particularly in the disjoint condition (S2 Fig).”

**2. For model recovery, a confusion matrix similar to the one used in Box 5 of Wilson & Collins (2019) would be much more informative than the current S2 table. Due to the importance of parameter values to the conclusion of the study, adding a parameter recovery analysis would be valuable and should not take excessive effort given the authors have already performed model recovery.**

Thank you for this:

- We have now updated our previous S2 Table into a confusion matrix (new S5 Fig), showing not only the percentage of the actual model being recovered (i.e. the diagonal and what was shown in the previous S2 Table) but also the off-diagonal, which describes where the confusion between models occurred. We have relabeled our figures and tables based on the order they are mentioned in the text.
- We note that we have already performed and reported parameter recovery, with the results shown in S7 Fig (previously S4 Fig).

**S5 Fig. Cross Model Recovery.** We generated 25 iterations of each model’s choice from the 142 participants' two conditions and experienced pair and reward trajectories where we refit them with each model’s best fitting parameters. Percentages show how often each model comparison metric (Akaike information criterion, AIC and Bayesian information criterion, BIC) recovers the true generated model, calculated as the proportion of successful recoveries across all simulations (25 iterations * 142 participants * 2 conditions). Due to better recovery of the hybrid model, we chose to continue model comparison using AIC.

**3. I appreciate the author’s clarification on the z-scoring approach. However, my concern is that it is adding something post-hoc into a model that is supposed to reflect cognitive mechanisms. Participants may or may not adapt their strategies to different magnitudes of rewards, but z-scoring based on previous five trials seems to be an arbitrary decision. The absolute scale of the reward could definitely matter within a single session, which the rolling z-scoring approach interferes with. Why not normalize the rewards based on the scale of the entire reward trajectory, or scale the betas based on the range of rewards? Whether or not z-scoring is applied shouldn’t affect model comparison (since the same trajectory should be fitted to by all models). If the concern is with comparing the model parameter across conditions, there should be statistical techniques that work for unbalanced sample sizes and the random walk condition can be included as a nuisance variable. As the author mentioned, despite the z-scoring procedure changing the beta weights significantly, this choice does not affect the parameter comparison conclusions qualitatively. I am still not convinced that the rolling z-scoring is necessary or justified and suggest the authors focus on results with either no z-scoring or other ways to remove the effect of reward scales.**

***“As the author mentioned, despite the z-scoring procedure changing the beta weights significantly, this choice does not affect the parameter comparison conclusions qualitatively”***

We apologize if we didn’t state this clearly, but we have not made that claim. To note, in the first round of revisions, we stated: “This would end up weighing some of the inverse temperatures based purely on the random walk a participant is in and make the random walks not comparable. Additionally, the experimental conditions will also make differences in our temperatures, due to the summation of reward to produce higher degrees of positive or negative prediction errors (such as an immediate 4 + delayed 4 to produce a summed 8). Given these three random walks, larger value differences will lead to a systematic reduction in our inverse temperature estimates. This might be akin to a regression equation where if you scale your data, then your coefficients will adjust as well.”

We understand both reviewers' concerns with our z-scoring approach and restate and further justify our reasoning. Additionally, if the reviewers are still concerned, we can provide a full analysis for each of the points below. In summary, z-scoring the value function removes some of the systematic biases in our parameter comparisons from the following problems:

1) Experimental
   a) Differences in the random walk of reward magnitudes will cause value differences that might scale the inverse temperature parameter and learning rate
   b) Differences in the random presentation of reward per individually experienced reward trajectory along with choice behavior (that should favor positive outcomes)
2) Model
   a) Differences in reward feedback conditions where summation (in the conjoint condition) can create both extra-large negative or positive prediction errors and/or completely nullify an object’s feedback
   b) Differences in the model’s value functions, as tabular representations hold both an immediate + 2-trial delayed dimension, whereas eligibility does not

***“Why not normalize the rewards based on the scale of the entire reward trajectory, or scale the betas based on the range of rewards?”***

In regard to post-hoc transformations, problems will emerge when computing the value difference at decision time, which will ultimately scale the strategy weight and trade-off with the learning rate. That is, the value differences are both heterogeneous with regard to the trial time and across the differing conditions. Different post-hoc normalizations, such as using the variance of value difference or other model parameters as a covariate, lead to vastly different conclusions, and we do not believe we have a good rationale for choosing a method of post-hoc normalization over another. However, finding the closed-form solution is difficult and we believe that reliance on simulation and model-fitting would provide the least bias. We performed an additional analysis to test the value-difference variance across participants with a Levene’s test for homogeneity of variance, separately for each experimental condition and random walk for the two models. This analysis shows that our scaling reduces the dispersion between various conditions, as can be seen in the figure below.

**S3 Fig. Comparison of conditions in value difference heterogeneity.** This figure compares the heterogeneity of model-predicted value differences, calculated as the variance across participants, between experimental conditions separately for the two scaling approaches (None; 5-Trial Z-Score) and the two models (Eligibility; Tabular). Levene’s test of heterogeneity shows that without scaling, there is significant heterogeneity in value difference for both eligibility, *F*(5, 278) = 3.99, *p* = .002, *η*^2^_p_ = .07, and tabular, *F*(5, 278) = 13.07, *p* < .001, *η*^2^_p_ = .19. However, scaling shows a non-significant effect of heterogeneity for eligibility, *F*(5, 278) = 1.29, *p* = .27, *η*^2^_p_ = .02, and a reduction in heterogeneity for tabular, *F*(5, 278) = 3.82, *p* = .002, *η*^2^_p_ = .06. The difference between scaling types was significant for eligibility, *F*(6, 556) = 12.42, *p* < .001 and tabular, *F*(6, 556) = 24.94, *p* < .001.

We have added this new figure to our supplementary document along with our thought process in the methods section (p.31, l.682): “We chose 5-trials to smooth around the lower end of memory recall, and that a form of adaptive normalization can appear to handle differing condition contexts (Hasselt et al., 2016; Pohlen et al., 2018). By normalizing the value function at decision time, the individual and hybrid models can be compared despite different value scales of the random walks and reward conditions (S3 Fig).” One might be able to see that both the random walk and experimental condition contribute to the variance of value differences. For example, the second random walk in conjoint is particularly high without scaling (green) but also appears moderately high in disjoint for the tabular model (blue).

For the reviewer’s reference, we have also plotted some example value difference trajectories of some example participants (N = 5) to highlight how the z-scoring method produces a similar scale.

***“Whether or not z-scoring is applied shouldn’t affect model comparison (since the same trajectory should be fitted to by all models). Participants may or may not adapt their strategies to different magnitudes of rewards, but z-scoring based on previous five trials seems to be an arbitrary decision.”***

Indeed, the reviewer is correct that while the scaling in the value function might affect model parameter values, the model-fit between experimental conditions did not change. We now show this in a supplementary table (S2 Table, also shown below), and in the methods section of the main text (p.31, l.687): “Although scaling the value function might affect model parameter values, the model-fit between experimental conditions did not change qualitatively (S2 Table)”

**S2 Table. Akaike information criterion (AIC) table for value transformation.**

| AIC | No scaling | | | | 5-trial Z-score scaling | | | |
| --- | --- | --- | --- | --- | --- | --- | --- | --- |
|  | Conjoint | Disjoint | V | p | Conjoint | Disjoint | V | p |
| Eligibility | 375.22 | 371.39 | 5725 | .19 | 375.55 | 374.86 | 5516 | .37 |
| Tabular | 373.9 | 360.81 | 6190 | .02 | 396.44 | 377.98 | 6647 | .001 |

This table displays Akaike Information Criterion (AIC) values in Conjoint and Disjoint conditions under two preprocessing approaches: raw value functions (no scaling) versus normalized value functions (5-trial Z-score scaling). The comparison demonstrates that value function scaling does not alter model fit patterns, as confirmed by Wilcoxon signed-rank tests comparing Conjoint and Disjoint for each model and each scaling approach.

***“However, my concern is that it is adding something post-hoc into a model that is supposed to reflect cognitive mechanisms.”***

We certainly agree that the absolute scale of reward might matter to the participant, and the cognitive interpretation of this is valuable. However, due to limitations of our statistical toolset, this is a non-trivial problem to address and remains an edge-case for a particular solution. The z-scoring method before decision time makes the contributions of different strategies **relative** to the participant’s current trial value function, which allows us to compare parameters across the models and different conditions. However, we lose understanding in the **absolute** comparison between the two models’ weight parameters and feedback conditions. We have changed our language throughout the manuscript to reflect relative strategy comparison and added additional sentences in our methods and discussion.

Methods (p.31, l.686), “This transformation handles statistical obstacles and allows a relative comparison between model parameters.”

Discussion (p.23, l.506), “We note that because the experimental conditions and random walk contained systematic deviations in reward magnitude, we chose to make our value function relative (i.e. scaled relative to the last 5 trials, see Methods for details and justification) to allow parameter comparison. Therefore, our analysis comparing strategy weights across conditions reflects scaling parameters that are relative to the participants’ recent history of experienced value. Despite this potential caveat, model-fitting metrics remained consistent regardless of scaling the value function, confirming the higher performance of the tabular model in the disjoint condition”

**4. For model comparison, it is necessary to include some information on the distribution of AICs to see if the differences in fit are actually significant. For example, this could be done with a rank-sum test or Bayesian Model Selection (Rigoux et al., 2014).**

We appreciate the reviewer’s suggestion, as we have been relying on an incorrect heuristic approach (ΔAIC < 2), and the suggestion for a Wilcoxon signed-rank or rank-sum test (depending on between or within-subject comparison) should supply a more formula test of comparison. We have modified Table 2, “P-values were calculated using paired Wilcoxon signed-rank tests compared to the best-fitting (indicated by -) model in each condition and highlighted in bold to indicate when a model couldn’t be statistically separated.” Along with modifying S1 Table, “We compared all models using a paired Wilcoxon signed-rank tests to the best-fitting (indicated by -) model in each condition.”

**Reviewer #2**

**The manuscript has been largely improved. However I still have a couple of lingering questions and think they are important to address before the acceptance of the paper.**

Thank you - we are happy to see that the reviewer finds the revised manuscript substantially improved and hope we have addressed the follow-up comments adequately.

**Major point:**

**(1) I am a bit concerned/confused by the value normalization after reading the response from the authors to reviewer 1. Why the value normalization leads to a change in # of subjects? The authors also argue that most of existing work does not have changed reward magnitude - which is not 100% true - there are a good amount of work in bandit task that generate reward from a gaussian distribution. I do agree with the authors that those work do not necessarily seek a cross-condition comparison though. I guess my confusion here is: what exactly does value normalization change? The authors have mentioned some possible scenarios, but I could use some help of some more concrete examples. Also, do the results from the current paper change drastically if value normalization is not applied?**

As mentioned in our response to reviewer 1, Z-scoring the value function allows us to remove some of the systematic biases in our parameter comparisons from the following problems:

1) Experimental
   a) Differences in the random walk of reward magnitudes will cause value differences that might scale the inverse temperature parameter and learning rate
   b) Differences in the random presentation of reward per individually experienced reward trajectory along with choice behavior (that should favor positive outcomes)
2) Model
   a) Differences in reward feedback conditions where summation (in the conjoint condition) can create both extra-large negative or positive prediction errors and/or completely nullify an object’s feedback
   b) Differences in the model’s value functions, as tabular representations hold both an immediate + 2-trial delayed dimension, whereas eligibility does not

***“Why the value normalization leads to a change in # of subjects?”***

Value normalization does not lead to a change in the number of subjects (N=142); however, there was a mismatch in session counts for the different random-walk and reward feedback conditions - that is the number of participants that experienced each random-walk is not exactly equal since the random walk was randomly assigned at the start of an experimental condition. This also means that a large majority of participants experienced different random walks in the two conditions.

We had this under the subheading Random-Walk Conditions from our supplementary document: “The random walk conditions were chosen due to the limitations of the online software and were randomized for each participant (28.17% of participants had the same random walk for both conditions). However, the sequence of decision pairs was totally random. Depending on choice from previous trials, participants could receive none, immediate, delayed, or a conjoint immediate and delayed reward.”

However, we decided to move this to the main document to remove any confusion (p.28, l.612): “At the start of each session, each participant was randomly assigned to one of the three random walks (due to limitations of the online software), meaning that some participants experienced the same random walk across both conditions (28.17%), while the majority experienced different random walks. ”

Additionally, we expanded our legend for Figure 1: “…Participants were randomly assigned to one of the three random walks at the beginning of each session (hence could experience different random walks in conjoint and disjoint condition) and encountered a random sequence of unique pairs (28 in total) in each session…”

***“The authors also argue that most of existing work does not have changed reward magnitude - which is not 100% true - there are a good amount of work in bandit task that generate reward from a gaussian distribution. I do agree with the authors that those work do not necessarily seek a cross-condition comparison though.”***

We generally agree with the sentiment here but also want to point out that the largest body of RL research on human participants has been on Rescorla-Wagner and temporal difference learning with simplified bandit tasks. Additionally, the bias extends further than just the reward function (problem #2a and #2b described above), and thus we thought it would be best to transform the value function at decision time rather than transforming the reward function (Kool et al., 2016; Konovalov & Krajbich, 2020). Although this is atypical in human experiments for non-stationary reward, we found that adaptive value normalization can help to reduce the variance in action-values and handle differing reward contexts (Hasselt et al., 2016; Pohlen et al., 2018).

***“I guess my confusion here is: what exactly does value normalization change? The authors have mentioned some possible scenarios, but I could use some help of some more concrete examples.”***

Problems will emerge when computing the value difference at decision time, which will ultimately scale the strategy weight and trade-off with the learning rate. That is, the value differences are both heterogeneous with regard to the trial time and across the differing conditions.  We performed a new analysis testing the value-difference variance across participants with Levene’s test for homogeneity of variance, separately for each experimental condition and random walk for the two models. Our scaling reduces the dispersion between various conditions, as can be seen in the figure below.

**S3 Fig. Comparison of conditions in value difference heterogeneity.** This figure compares the heterogeneity of model-predicted value differences, calculated as the variance across participants, between experimental conditions separately for the two scaling approaches (None; 5-Trial Z-Score) and the two models (Eligibility; Tabular). Levene’s test of heterogeneity shows that without scaling, there is significant heterogeneity in value difference for both eligibility, *F*(5, 278) = 3.99, *p* = .002, *η*^2^_p_ = .07, and tabular, *F*(5, 278) = 13.07, *p* < .001, *η*^2^_p_ = .19. However, scaling shows a non-significant effect of heterogeneity for eligibility, *F*(5, 278) = 1.29, *p* = .27, *η*^2^_p_ = .02, and a reduction in heterogeneity for tabular, *F*(5, 278) = 3.82, *p* = .002, *η*^2^_p_ = .06. The difference between scaling types was significant for eligibility, *F*(6, 556) = 12.42, *p* < .001 and tabular, *F*(6, 556) = 24.94, *p* < .001.

We have added this new figure to our supplementary document along with our thought process in the methods section (p.31, l.682): “We chose 5-trials to smooth around the lower end of memory recall, and that a form of adaptive normalization can appear to handle differing condition contexts (Hasselt et al., 2016; Pohlen et al., 2018). By normalizing the value function at decision time, the individual and hybrid models can be compared despite different value scales of the random walks and reward conditions (S3 Fig).” One might be able to see that both the random walk and experimental condition contribute to the variance of value differences. For example, the second random walk in conjoint is particularly high without scaling (green) but also appears moderately high in disjoint for the tabular model (blue).

For the reviewer’s reference, we also have plotted some example value difference trajectories of some of our participants (N = 5) to highlight how the z-scoring method produces a similar scale.

***“Also, do the results from the current paper change drastically if value normalization is not applied?”***

Without value normalization, parameter values - particularly strategy weights - are no longer comparable, but our model-fitting metrics do not change. We now show this in a supplementary table (S2 Table, also shown below), and in the methods section of the main text (p.31, l.687): “Although scaling the value function might affect model parameter values, the model-fit between experimental conditions did not change qualitatively (S2 Table)”

**S2 Table. Akaike information criterion (AIC) table for value transformation.**

| AIC | No scaling | | | | 5-trial Z-score scaling | | | |
| --- | --- | --- | --- | --- | --- | --- | --- | --- |
|  | Conjoint | Disjoint | V | p | Conjoint | Disjoint | V | p |
| Eligibility | 375.22 | 371.39 | 5725 | .19 | 375.55 | 374.86 | 5516 | .37 |
| Tabular | 373.9 | 360.81 | 6190 | .02 | 396.44 | 377.98 | 6647 | .001 |

This table displays Akaike Information Criterion (AIC) values in Conjoint and Disjoint conditions under two preprocessing approaches: raw value functions (no scaling) versus normalized value functions (5-trial Z-score scaling). The comparison demonstrates that value function scaling does not alter model fit patterns, as confirmed by Wilcoxon signed-rank tests comparing Conjoint and Disjoint for each model and each scaling approach.

**(2) hybrid model:I understand that the authors try to make the case that given different experiment conditions - that is, different information uncertainty - people may adopt different exploration strategy (or put different weights on a set of strategies). In that case, would it be more intuitive in data analysis to compute weight as the ratio of beta for two strategies and see how that changes across conditions? Relatedly, on an implementation level, are these two betas fit once, and then for the single-strategy models the authors use one of the fitted beta and fix the other one to be 0? Or do they do three separate fittings for three models?**

The reviewer makes a valid point on using a beta-ratio; however, our experimental design, as well as Tanaka et al.’s (2007) original design, was not aimed at being competitive between learning systems. Thus, we believe that our approach here (with the two beta models) lends itself more to a mixture model with the eligibility model and tabular model influence, rather than a competitive comparison between the two models.

“***Relatedly, on an implementation level,* *are these two betas fit once, and then for the single-strategy models the authors use one of the fitted beta and fix the other one to be 0? Or do they do three separate fittings for three models?”***)

The implementation could have been further clarified in the manuscript - we indeed performed three separate fittings for the three models. We have now clarified this in the methods (p.32, l.705): “Each of the three models (eligibility, tabular, and hybrid) was optimized separately for each of the experimental conditions (conjoint, disjoint) using an evolutionary strategy (R package ‘DEoptim’).”

**Minor points:**

**(1) For model recovery (Table S2), how many total simulations was run?**

In total there were 25 (iterations) * 142 (participants) * 2 (conditions) simulations ran. We have changed our model recovery table to a confusion matrix (now S5 Fig), following Reviewer #1’s suggestion, and added the previous note in the Figure caption: “We generated 25 iterations of each model’s choice from the 142 participants' two conditions and experienced pair and reward trajectories where we refit them with each model’s best fitting parameters. Percentages show how often each model comparison metric (Akaike information criterion, AIC and Bayesian information criterion, BIC) recovers the true generated model, calculated as the proportion of successful recoveries across all simulations (25 iterations * 142 participants * 2 conditions). Due to better recovery of the hybrid model, we chose to continue model comparison using AIC.”

**(2) when interpreting model comparison results, the authors refer to delta AIC = 2 as a threshold for meaningful difference. Is this criterion obtained from any book/paper citation?**

This is a common heuristic to compare models that may have originated from Burnham and Anderson (2002; 2004). However, it would appear that it is commonly misused, as an increase in a free parameter will approximately decrease AIC by 2, so this is mainly for comparison between models of differing parameter sizes (Arnold, 2009).

Following Reviewer #1’s suggestion, we are now using a Wilcoxon signed-rank or rank-sum test (depending on within-subject or between-subject comparison) for a formal test of comparison. We have modified Table 2 and S1 Table to show this.

**(3) on p6: 'This leads to our final questions: whether reward learning under temporal uncertainty can be empirically solved by a mixture of prospective and retrospective strategies' <- Should this be information uncertainty given the updated terminology?**

We would argue that we are still measuring reward learning under **temporal** uncertainty (the process that is being captured overall in our task), but we use **information** uncertainty as an external manipulation. We have expanded the sentence in our manuscript, (p.6, l.129): “This leads to our final questions: whether reward learning under temporal uncertainty can be empirically solved by a mixture of prospective and retrospective strategies, and whether we can experimentally shift control to the most reliable strategy by manipulating information uncertainty through the observability of the reward function.”

**(4) the result section when describing Fig 3B: would be helpful if the authors can remind the readers that they are using the overlapped confidence intervals as an indicator for model performance - and maybe report the CI stats for some important comparisons. the current language (e.g., ‘follow participants behavior’, ‘had mixed patterns’) is a little vague for the discussion section.**

We are in agreement with the suggestion, and we have added an additional sentence on using overlapping confidence intervals as an indicator of model performance. We also decided to provide the confidence intervals for the **difference** between positive and negative stay probability when describing Figure 3B in the results section, as these might be some of the more important comparisons to highlight for the reader. The updated description mentioning those CIs for stay probability following positive versus negative outcomes now reads as follows (p.12, l.253): “To better understand how our models mapped with participants’ behavior, we inspected the extent to which the 95% CI of overlapped between the model predictions and the participants’ data. Figure 3B shows margin overlaps with text annotations, while differences between positive and negative rewards are reported with 95% CIs in the text. The three-way interaction shows that in the conjoint condition, tabular (Δ_+-_0F 95% CI [0, .12], Δ_+-_2F 95% CI [.05, .16]), and the eligibility model (Δ_+-_0F 95% CI [0, .12], Δ_+-_2F 95% [.0, .1]), were similar and both overlapping with participants’ means (Δ_+-_0F 95% CI [.04, .18], Δ_+-_2F 95% [.06, .17]). However, in the disjoint condition, the tabular model mapped to participants better than eligibility for 2F (Δ_+-_2F 95% CI tabular [.14, .26], participants [.18, .29], eligibility [.02, .13]), while the opposite pattern tended to emerge for 0F (Δ_+-_0F 95% CI tabular [-.04, .09], participants [.02, .15], eligibility [0, .14]).”.

Additionally, we have corrected an error where we accidentally included an immediate object in the analysis, but this does not change our results. Lastly, we have bootstrapped our standard errors to account for the 25-iterations of model choice behavior to make the graph more accurate. We have pasted the new graph from Figure 3B below.

**Fig 3. Behavioral signatures of learning and model predictions.** ...(B) The probability of maintaining (i.e. staying on) a delayed choice three trials later were modeled using multilevel logistic regression, as a function of condition (Conjoint vs Disjoint), time (Immediate, 0F or two-trial forward, 2F), and reward (positive, + or negative, -), as well as their interactions (equation 1). This regression was run on data from participants (red), as well as data generated by the tabular model (green) and data generated by the eligibility model (blue) for comparison. The logistic regression’s estimated marginal means are displayed, accompanied by 95% confidence intervals for each condition. Text displays when participants’ estimated confidence intervals overlap with eligibility [E,-], tabular [-,T], or both [E,T]...

**(5) I appreciate the additional analysis related to task difficulty (Fig S1). However I am pretty confused by what (B) and (C) means. Why use AIC as the DV? for (B), in caption it suggests negative coef for IVs, but on the graph it seems that the lines have positive slope? In the caption the authors mentioned ‘make people more stochasticity and less consistent’ - is this based on interpreting smaller AIC or something else?**

We generally used AIC as a metric of model fit to participant choice, wherein higher values would indicate lower predictive accuracy of participants’ choices; however, we have decided to replace this with the actual negative log-likelihood values, which might reduce any confusion. Higher negative log-likelihood also indicates lower performance of the model in explaining participants’ choices. We have now copied the updated S1 Figure below, and substantially updated the figure caption to make it clearer.

For panel (B), we apologize and have corrected the regression to perform a three-way interaction (along with replacing AIC with log-likelihood). With regard to making people more stochastic and less consistent, the reviewer is correct that this language is confusing; rather, it was meant to illustrate that the participants’ choices were less consistent with the model predictions as difficulty increases. The purpose was to identify whether the effect of difficulty on model performance (negative log-likelihood) was systematic or whether it varied depending on the model, which could suggest a role for difficulty in relying on one model versus the other.

For (C), we performed a similar analysis with a more “objective” measure of difficulty (i.e. not self-report), namely the average rate at which rewards change from one trial to the next, to show that findings hold with two different proxies for difficulty.

**S1 Fig. Task difficulty.** (A). Perceived task difficulty ratings collected at the end of the study were not significantly different between our two groups of participants (those who started with the conjoint condition vs those who started with the disjoint condition, *t*(140) = -0.12, *p* = .9). (B-C). Effect of task difficulty on model performance. We used negative log-likelihood as a metric of predictive accuracy of our model to participant data, with higher values indicating lower predictive accuracy. (B) Using linear regression predicting negative log-likelihood from perceived difficulty, model, and reward condition showed no three-way interaction (*p* = .62), nor any two-way interactions (all *p* > .27). There were significant main effects of perceived difficulty, *t*(564) = 2.31, *p* = .02 and model, *t*(564) = 2.13, *p* = .03, but no effect of reward condition, *t*(564) = -1.7, *p* = .09. This suggests that participants’ choices are less consistent with either model as perceived task difficulty increases, rather than difficulty selectively impacting the tendency of participants to rely on one model over the other. (C). A similar analysis was run with the average rate of reward change per participant as a proxy for difficulty (rather than the self-reported perceived difficulty). Reward change was calculated as 0 when the selected object’s reward value did not change from the last time it was previously selected and 1 otherwise, with the assumption that higher reward change rate is associated with higher task difficulty. We fit a linear regression predicting negative log-likelihood values from an interaction between model, reward change rate, and reward condition. Once again, there was no three-way interaction (*p* = .87), nor any two-way interactions (all *p* > .57). There were significant main effects of reward change rate, *t*(564) = 9.39, *p* < .001, model, *t*(564) = 2.28, *p* = .02, and reward condition, *t*(564) = -2.74, *p* = .006. Thus, although difficulty does appear to negatively impact model fit overall, this effect did not vary according to the model or the reward condition.
