# Supplementary material for: Information uncertainty influences learning strategy from sequentially delayed rewards
Source: PLoS Comput Biol. 2026 Feb 2;22(2):e1013879. doi: 10.1371/journal.pcbi.1013879 (PMC12885371; doi:10.1371/journal.pcbi.1013879)
Supplement: Attachment — Submitted filename: Response to Reviewers_v3.docx [file pcbi.1013879.s017.docx]

Dear Reviewers,

Thank you for reviewing our revised manuscript, “*Information uncertainty influences learning strategy from sequentially delayed rewards*” (PCOMPBIOL-D-25-00171) and for your questions and concerns. We thank the reviewers for their additional support in improving the manuscript.

We have clarified the last comments from Reviewer #1 by (1) providing a new simulation analysis (new S1 Fig) that demonstrates the behavioral dissociation between the two models as predicted by theoretical accounts and our depiction in Figure 2C, and (2) showing that our findings (model fits across conditions – updated S2 Table - and parameter distributions – new S5 Fig) hold under different normalization schemes (comparing our 5-trial scaling window, to 1-trial, 3-trial, 7-trials, and all 336 trials).

We have added your comments below in bold (with subpoints in bold-italics) followed by our responses with page and line number for any changes within the manuscript.

With these additional revisions, we hope the manuscript will be suitable for publication at *PLoS Computational Biology*.

Again, we appreciate the time and consideration from both reviewers, and we look forward to hearing from you.

Sincerely,

Caroline J. Charpentier, PhD	Sean R. Maulhardt, MA

Email: [ccharpen@umd.edu](mailto:ccharpen@umd.edu)	Email: [smaulhar@umd.edu](mailto:smaulhar@umd.edu)

**Reviewer #1**

**Reviewer #1: I really appreciate the thorough analyses conducted in the revision, especially in model recovery and comparison, as well as the authors’ effort in clarifying points of confusion. I believe most of my concerns have been addressed. Here are a few final lingering questions:**

We appreciate the comments on the manuscript and believe that our additional revisions further clarify and support our findings.

**1. I’m confused at why the eligibility and tabular models display such similar behavior in the WSLS adherence plot. As shown in the illustration in Figure 2C, these models should have qualitatively distinct credit assignment behavior. Specifically, how could reward at lag 0 lead the eligibility model to accurately repeat object at lag 2, but not repeat object at lag 1? It makes sense that there is no real systematic relationship between outcomes at 0 and the choice at lag 1 and 3. However, doesn’t the eligibility model predict that participants will nonetheless falsely associate that outcome with those choices, especially choice at lag 1 due to it being temporally closer to the outcome at lag 0? While I think these analyses do not necessarily undermine the author’s claims, the results would be much clearer and stronger if the authors can directly demonstrate the behavior predicted in Fig 2C in the data and in the models.**

We believe our confusion resided in how the behavioral signature materializes over the course of the experiment (WSLS adherence or S3 Fig). Assuming the models carried no memory of previous values, we can display the difference of credit assignment mechanism at trial time. To achieve this, we used the simulation data from the participants’ best fitting parameters and gathered the value function by reward condition and model (eligibility and tabular) to calculate the credit assignment difference for different time lags. We drew the values (value_t_^st-t3^) of each current trial’s (t) selection (s^t^) and 3 of the trials’ previous selections (s^t – t3^). We then used this time vector to take the difference from the value function 1 trial in the future (value_t+1_^st-t3^ - value_t_^st-t3^). Doing this allowed us to get credit applied to each of the previously chosen options regardless of the option’s memory of their current value function. We excluded selection windows (current, t-1, t-2, t-3) that contained repeated selections because repeated selections only refreshed eligibility traces without triggering credit assignment. This allowed us to identify the model difference in credit assigned along a 4-trial window for each trial in the participant’s experienced trajectory.

We add this into the results section, **p.9 l.203**, “We provide a graphical representation in Fig 2 and recreate the credit assignment mechanisms from model-simulated data (S1 Fig)”, and the methods section, **p.30, l.669** “These credit assignment mechanisms lead to distinct signatures in model simulations (S1 Fig).” As such, we display our new figure in the supplemental.

**S1 Fig. Model Credit Assignment Signatures.** We display our two models (eligibility and tabular) credit assignment mechanism using participants’ best fitting parameters to simulate the following value calculation. For each trial (starting on trial 4), we calculated the credit difference, which corresponds to the value update between the current and the next trial for the current trial selection (Time 1). Credit difference values for Time 2 to 4 represent the same value update calculation, but for the options selected in trials t-1, t-2, and t-3 respectively. We filtered out selection windows with repeated choices to identify the gradient of credit assignment to the last few selections. Values are displayed as a function of prediction error valence (whether the outcome of the current trial is positive or negative), condition (conjoint or disjoint), and model (eligibility or tabular). These simulations align with the prediction of main text Figure 2C, demonstrating the diminishing gradient in credit assignment for the eligibility trace model and the ‘jump’ or discard of systematically irrelevant delays (t-1 and t-3) for the tabular model

We appreciate the reviewer’s additional comments and hope this demonstration aligns with the credit signature in Figure 2C.

**2. I understand the author’s rationale for normalizing the values, as different random walk reward trajectories will lead to different scales of values, which then leads to differences in the scale of model parameters that may reflect artifacts of model fitting procedure and not psychologically meaningful parameters. It is very reasonable that some normalization is needed to allow for cross-session comparison. However, the choice of dynamic z-scoring by 5 trial windows is still arbitrary, especially compared to a simple global scaling of the entire value trajectory. As participants make different choices that lead to different outcomes, their Q-values will naturally diverge with more trials, and I don’t believe it is necessary to use a dynamic z-scoring procedure to fully remove this heterogeneity. If different normalization strategies lead to different results, then it should be more important to show that the main conclusion hold under different normalization schemes.**

We understand that the point of the 5-trial normalization seems arbitrary; however, we believed that using a larger window of normalization would be influenced from large prediction errors (from summed rewards or random walks). The reviewer does make a valid point, *“If different normalization strategies lead to different results, then it should be more important to show that the main conclusion hold under different normalization schemes”*. To address this, we re-ran our optimization with other dynamic z-scoring windows with 1, 3, 5 (original), 7, and 336-trials as the window. First, we inspected the AICs of the different scaling approaches in an expanded S2 Table.

**Expanded-S2 Table. Akaike information criterion (AIC) table comparing value normalization schemes.**

| AIC | No scaling | | | | 5-trial Z-score scaling | | | |
| --- | --- | --- | --- | --- | --- | --- | --- | --- |
|  | Conjoint | Disjoint | V | p | Conjoint | Disjoint | V | p |
| Eligibility | 375.22 | 371.39 | 5725 | .19 | 375.55 | 374.86 | 5516 | .37 |
| Tabular | 373.9 | 360.81 | 6190 | .02 | 396.44 | 377.98 | 6647 | .001 |
|  | 1-trial Z-score scaling | | | | 3-trial Z-score scaling | | | |
|  | Conjoint | Disjoint | V | p | Conjoint | Disjoint | V | p |
| Eligibility | 374.94 | 374.21 | 5526 | .36 | 375.34 | 374.61 | 5525 | .36 |
| Tabular | 375.29 | 376.71 | 6637 | .001 | 395.96 | 377.34 | 6655 | .001 |
|  | 7-trial Z-score scaling | | | | 336-trial Z-score scaling | | | |
|  | Conjoint | Disjoint | V | p | Conjoint | Disjoint | V | P |
| Eligibility | 375.73 | 375.06 | 5534 | .35 | 373.52 | 373.81 | 5409 | .5 |
| Tabular | 396.79 | 378.53 | 6633 | .002 | 396.93 | 376.62 | 6701 | <.001 |

To test whether our main conclusion is held under different normalization schemes, we compared our theoretically motivated 5-trial scaling window with other scaling schemes (no scaling, 1-, 3- and 7-trial scaling window, and scaling across the entire task). Displaying Akaike Information Criterion (AIC) values for Conjoint and Disjoint conditions under two preprocessing approaches: raw value functions (no scaling) versus normalized value functions (5-trial Z-score scaling). The comparison demonstrates that value scaling does not alter model fit patterns, as confirmed by Wilcoxon signed-rank tests (V) comparing Conjoint and Disjoint for each model and each scaling approach.

Given that the directionality in AIC difference between the two reward conditions did not change for either model, we also examined the possibility that parameter distributions might differ under different normalization schemes. We display this below along with performing regression as a function of parameter value and the interaction between scaling method, parameter, and reward condition. We use the ‘emmeans’ package to perform pairwise comparisons between the scaling methods.

**S5 Fig. Comparison of Z-trial dynamic value scaling.** Using linear regression, we predicted each parameter value as an interaction between trial-scale, parameter, and reward condition. We then use the ‘emmeans’ package to perform pairwise comparisons between the scaling methods. The only significant differences was found with no-scaling and all other dynamic Z-trial methods, with 1-trial, b = .1, SE = .014, t(10152) = 7.27, p < .001, 3-trial, b = .107, SE = .014, t(10152) = 7.76, p < .001, 5-trial, b = .108, SE = .014, t(10152) = 7.84, p < .001, 7-trial, b = .11, SE = .014, t(10152) = 7.96, p < .001, and 336-trial, b = .136, SE = .014, t(10152) = 7.96, p < .001. The 336-trial dynamic Z-scaling was the most different from the other scaling methods but did not reach significant for 1-trial, p = .1, 3-trial, p = .29, 5-trial, p = .34, or 7-trial, p = .411.

As one might expect (and discussed in our previous revisions), the largest differences appeared for the no-scale condition with contrasts showing significant differences to all other dynamic Z-scoring methods. We added a sentence in the methods to mention these two analyses, **p.31, l.688**, “We tested alternative normalization trial windows, which led to similar results in AIC (S2 Table) as well as non-significant changes in parameter distributions (S5 Fig). Specifically, our finding of reduced tabular use in the conjoint condition was independent of the normalization scheme used (S2 Table). As for parameter distributions, outside of expected differences in weight parameters when no scaling is applied, all scaling methods yielded similar parameter values (greatest difference found between 1-trial and 336-trial, p = .1).”

**Looking at reviewer #2’s comment, I also wonder why the authors did not look at the ratio of each strategy’s parameters in the hybrid model. Since both parameters are fit to the same random walk trajectory, wouldn’t looking at their ratio remove the effect of reward scales? If so, wouldn’t comparing such ratios remove the need for any normalization?**

We appreciate the comment and wish to further clarify our reason for not using the ratio-method. From our last revision we stated that both models implement differing mechanisms and this would cause systematic deviations in the two reward conditions, specifically: “Differences in reward feedback conditions where summation (in the conjoint condition) can create both extra-large negative or positive prediction errors and/or completely nullify an object’s feedback” and “Differences in the model’s value functions, as tabular representations hold both an immediate + 2-trial delayed dimension, whereas eligibility does not”.

Additionally, a ratio between the two betas would be difficult to interpret, such as “Does a ratio of .5 reflect a reduction in tabular or are they equivalent as the value functions have different scales?” or “Does a change in the ratio between Conjoint and Disjoint mean a change in Tabular weight only, in Eligibility weight only, or in the two models weights changing in different directions?”. Furthermore, one could use a single parameter for the ratio but then identifiability might become an issue, such as a two-to-one ratio being represented under differing scales (IE: 2/1 or .2/.1). As such, we believe the two-beta model (now combined with the demonstration of robustness to different normalization schemes) is sufficient to answer our questions, provide more mechanistic insights, and help to pave the way for more ecologically valid designs and modeling.

**Reviewer #2: The authors have addressed all my concerns. Just a quick note that please also remember to update the osf repo to reflect the new analysis that has been done. Thanks for the hard work.**

We appreciate the reviewers’ comments and thank them for their support of our manuscript; we are currently working to update the OSF repository and will ensure all the materials uploaded match the finalized version of the manuscript.
